# 1475. A Retrospective Study Analyzing Recurrent LVAD Driveline Infections

**DOI:** 10.1093/ofid/ofad500.1311

**Published:** 2023-11-27

**Authors:** Kathy Tin, Muhammad K Malik, Maressa Santarossa, Mingxi Yu, Max Liebo, Nina M Clark, Gail Reid

**Affiliations:** Loyola University Medical Center, Chicago, Illinois; Loyola University Medical Center, Chicago, Illinois; Loyola University Medical Center, Chicago, Illinois; Loyola University Medical Center, Chicago, Illinois; Loyola University Medical Center, Chicago, Illinois; Loyola University Chicago Stritch School of Medicine, Maywood, IL; Loyola University Chicago, Stritch School of Medicine, Maywood, IL

## Abstract

**Background:**

Left ventricular assist devices (LVADs) are an advanced form of heart failure therapy for patients who failed conventional medical therapy. LVAD patients are at increased risk for developing infections due to the presence of foreign hardware. Infection of the LVAD driveline (DL), the connection between the electrical cable and air vent to an external site, is the most common type of LVAD infection. Due to limited data available, this study sought to investigate outcomes of recurrent LVAD driveline infections (DLI).

**Methods:**

This was a retrospective chart review study at Loyola University Medical Center of patients with LVAD driveline infections over 14 years. The primary outcome was days to recurrent DLI. Recurrent DLI was defined as any antibiotic alteration due to infection by a new organism, relapse with the same organism, and/or clinical signs or symptoms of worsening infection. Secondary endpoints included recurrent infection after surgical management, abscess development, and post-heart transplant (HT) infection.

**Results:**

35 of 49 patients (71.4%) developed a recurrent DLI with a mean of 173 days (range 14-820 days) to recurrent infection. The most common organisms were methicillin sensitive *Staphylococcus aureus, Pseudomonas aeruginosa*, coagulase negative *Staphylococcus*, and *Corynebacterium* species. 4 patients with recurrent DLI developed local abscesses. 7 patients with recurrent DLI (20.0%) underwent DL debridement and/or revision, and 4 of these patients (57.1%) developed recurrent DLI afterward. 1 of the remaining 3 patients underwent HT 198 days after the DL procedure. 10 of the 35 patients with recurrent DLI underwent HT and 4 developed infections within 90 days of transplant; 2 were due to the same organism that caused the DLI. One of these had a retained portion of the DL and the other had a retained ICD lead. There were also 2 sternal wound infections of unknown cause.

**Conclusion:**

Most patients with DLI developed recurrent infection. Additionally, DL debridement/revision was not successful in curing infection in most patients. In some cases, recurrent DLI may have been due to antibiotic non-adherence. Other than patients with retained device components, those who underwent a heart transplant did not have infections 90 days post-transplant caused by the DLI organism.

**Disclosures:**

**Kathy Tin, D.O.**, Merck: Stocks/Bonds|Novavax: Stocks/Bonds|Pfizer: Stocks/Bonds

